# Medical and Physical Intelligence

**Published:** 1822-02

**Authors:** 


### Medical and Physical Intelligence.

### 1. *Gastrotomy.*

A YOUNG man accidentally swallowed a silver fork, eight inches in length, in consequence of which the most threatening symptoms ensued, and the greatest alarm was entertained. Mr. Renaud, an able surgeon at Romans, (department of the Drôme,) with a view to afford the only assistance in his power to the patient, undertook the operation of gastrotomy; which he performed in the presence of several physicians of the Faculty of Medicine of Paris and Montpellier, and succeeded, with much dexterity, in extracting the fork. The young man is considered out of danger, and was expected to recover in a short time.—*Tablettes Universelles*, Oct. 1821, p. 61.

### 2. *New Instrument for Cataract.*

Professor Gibson, of the University of Pennsylvania, has lately invented an instrument, exceedingly well calculated for cutting to pieces the crystalline lens, in all cases of cataract. It is well known that the knives of Saunders, Sir William Adams, and other oculists, are defective, in not being sufficiently strong and sharp to divide the lens when it happens to be of a very hard texture. Under such circumstances, the cataract is soon dislocated, in the attempt to divide it by the knife, and is either depressed into the vitreous humour, or else enucleated from its capsule. To obviate these inconveniences, Dr. Gibson has contrived a scissor so delicate, as hardly to exceed, in size, the iris-knife of Sir William Adams, and, at the same time, so strong and sharp as to cut, with ease, the most solid and compact lens and capsule, without injuring, in the slightest degree, any part of the eye. These scissors are formed upon the principle of Mr. Woollaston's scissors, used for common purposes, with the edge so constructed as to operate like a knife. On this account, the instrument perforates the coats of the eye with the utmost facility, and, when introduced, the blades can be opened to a certain extent, so as to cut the lens to pieces, without bruising it or any other part; the necessary effect of scissars, as they are usually made. This instrument possesses another advantage: the lens is supported in its natural situation during the operation, by having one blade behind, and the other before it, so that it may be cut to pieces *in situ*, and its remains afterwards forced, by the shut blades, into the anterior chamber, for dissolution. In this way the operation of Sanders and Adams may be performed with great effect, and without that necessity for repetition which so often occurs when the common instruments are employed. Dr. Gibson has made trials with the instrument, sufficient to convince him of its decided superiority over every other used for the same purpose.—*Philadelphia Journal.*

### 3. *Dispensary in Switzerland.*

It is, perhaps, not generally known, that a medical Dispensary, in imitation of those of England, was opened at Geneva, about twelve months since, by Dr. Gosse, Dr. Prevost, and Mr. Dupin, surgeon. The house of this institution is situated in a convenient part of the town; and the patients are received three days in the week; those who are too ill to attend being visited at their own habitations. The subscribers are divided into classes, paying twelve, twenty-four, and thirty-six francs annually; enjoying privileges proportionate to their subscription. Dr. Gosse is well known for his philanthropic exertions during the scarcity which prevailed, in Switzerland, in the year 1817. —*Revue Encyclopedique,* Nov. 1821.

### 4. *Royal Atheneum at Paris.*

The various courses of Lectures, to be delivered at this institution in the year 1822, being the thirty-seventh year of its foundation, have recently begun, in the following order, and by the following professors:

1. Experimental Philosophy, by M. Pouillet,

3. Chemistry ................ M. Robiquet,

3. Zoology .................. M. Blainville,

4. Anatomy and Physiology.. M. Magendie,

5. Astronomy.............. M. Francoeur,

6. Geology................ M. Constant Prevost,

7. Literature............. M. Lingay,

8. General Philosophy..... M. Azaïs.

—*Revue Encyclopedique*, Nov. 1821.

### 5. *Latinity of Sydenham.*

A correspondent, from Worcester, asks two plain questions of our readers:—Did Dr. Sydenham translate his works into Latin himself? Are there any proofs positive that he did so?—Our correspondent's reasons for doubting, (for he, who asks a question, doubts,) are these:—In Ward's Lives of the Professors of Gresham College, it is said, that "Sydenham dedicated his works to Dr. Mapletoft, and that that physician did, by his desire, translate them into Latin; but not all; for some were translated by a Mr. Gilbert Havers, of Trinity College, Cambridge, a student of physic." And again, in another author, we find this passage, in speaking of Sydenham: "It is observable, that his *Processus Integri*, published after his death, discovers, alone, more skill in Latin than is commonly ascribed to him." On the other hand, our correspondent admits, that Sir Hans Sloane, who was intimately acquainted with Sydenham, bore testimony of that great physician's intimate knowledge of the language of Cicero.—Here is food for the skilled in ancient lore; and we shall be happy to see the subject properly discussed.

### 6. *American Medical Literature.*

In the Number of the Philadelphia Journal for November, which has just reached us, we have remarked, that out of twenty-five articles—of which the department of that Journal entitled Medical and Philosophical Intelligence, consists—twenty-three are copied, *literatim*, from those Numbers of the *London Medical Intelligencer* which were edited by the person who has now the honour of conducting the London Medical and Physical Journal. It is gratifying to see this mark of predilection on the part of our transatlantic brethren. The two remaining articles are taken from the Edinburgh Medical Journal and from Dr. Johnson's Review.

### 7. *Thomsonite.*

This is the name given to a new mineral substance found in the neighbourhood of Norwich, by Mr. R. Squires, who has sent a detailed description of the mineral to the editor of the Annals of Philosophy. From the analysis it would appear, that the substance is a new variety of crystallized magnesian carbonate of lime. It is generally of a snow white, sometimes yellowish white, and, in a few specimens, passes into asparagus green. It occurs crystallized in four sided prisms, terminated by flat irregular sided pyramids, levelled at the extremities. It appears to be composed of lime 30, magnesia 19, carbonic acid 38, silica 6, potash and water, including loss, 6 = 100. —*Annals of Philos. Oct.* 1821.

### 8. *Infantile Polysarcia.*

In a country village, about twelve miles from Bristol, there is a child, whose present appearance bids fair to equal, if not surpass, the wonderful prodigy at Leicester. The child is only six months old, measures seven inches round the arm-wrist, and nine inches three-quarters round the small of the leg; he is supposed to weigh between forty and fifty pounds. He is quite a burden to be held long in the arms; appears lively and healthy, the flesh firm and hard; and is asserted, by his mother, to have commenced eating animal food at a fortnight old. He is the child of a country shoe-maker. His face and all his limbs are in proportion; but he does not yet walk.

### 9. *Curious Mode of calculating the Duration of a Man's Life.*

The length of a man's life may be estimated by the number of pulsations which he has strength to perform. Thus, allowing seventy years for the common age of man, and sixty pulses in a minute; the common measure of pulses, in his whole life, would amount to 2,207,520,000; but, if, by intemperance, he forces his blood into a more rapid motion, so as to give seventy-five pulses in a minute, the same number of pulses would be completed in fifty-six years; consequently, his life would be reduced fourteen years.

### 10. *Pulmonary Worms.*

Mr. Rogers has made a discovery, which may ultimately prove of some importance. He says, "in making some microscopic experiments with small spherules of very high magnifying powers, I have observed that the matter, or pus, expectorated in a certain stage of pulmonary consumption, is actually filled with multitudes of minute worms; the forms of which, in their evolutions from the surrounding mucus, are so distinctly seen, as to obviate all doubt of their identity with living animalculi. I am induced to hope that an attentive consideration of the subject, by those professionally competent to its investigation, may tend to throw a light on the (at present) inscrutable cause of this malady. Is it unreasonable to regard these worms, the existence of which is indisputable, as forming the concomitant cause of consumption? And may not the irritation, consequent on the motions and gnawings of so many thousands of these vermin in so sensitive an organ, account for the restless excitability of the parts, the ulceration, and some other incidental symptoms? I have observed the worms to be killed, while under the microscope, by exposure to a particular kind of gas; which it might be imprudent, in this stage of the inquiry, to name."

### 11. *Pearl found in the Human Body.*

Wesley, the celebrated founder of Methodism, contracted a hydrocele, and submitted to the operation for its cure when he was seventy-one years of age. In Southey's Life of Wesley, (vol. ii. p. 539,) we find the following note, extracted from the journal of the preacher. "Mr. Wathen performed the operation, and drew off something more than a half-pint of a thin, yellow, transparent water: with this came out (to his no small surprise,) a *pearl* of the size of a small shot, which he supposed might be one cause of the disorder, by occasioning a conflux of humours to the part."—*Journal*, xvii. p. 8.

12. *Zoological Collection, from Captain Franklin.*

At the last meeting of the Linnean Society, Mr. Joseph Sabine read a paper, containing the description of twelve species of quadrupeds, and fifty-six species of birds, sent home by Captain Franklin and Dr. Richardson, forming the land expedition to the Polar Seas. Mr. Sabine had received the specimens from Lord Bathurst, of the Colonial Office, accompanied by an order that they should be accurately described, with a view to their being published in the Transactions of the Society, and the specimens deposited, afterwards, in the British Museum.

13. *Return of* Dr. Laennec *to Paris.*

We are happy to have it in our power to announce the complete recovery, and return to the capital, of this truly eminent physician and pathologist. His unremitting attendance to the duties of his hospital, and the arduous task he had accomplished, and of which the public have witnessed the result, had so far impaired his health and constitution, as to oblige him to retire into the country after the publication of his classical work. It must be gratifying to the friends of medical science to know that he is now perfectly recovered, and that he intends resuming the particular object of his studies.

14. *Prophetic Medicine.*

Dr. Most, of Hamburgh, in an elaborate work on the History and Nature of Influenzas, published in 1820, of which we have given a short account in another part of the present Number, has predicted the re-appearance of that disorder in the course of the present year. His prediction is, principally, founded on this singular coincidence,—that the epidemic influenzas, which have hitherto appeared in Europe, and of which we possess an accurate knowledge, took place at an interval of twenty years between each epidemic; the last having occurred in 1802.

15. *Treatment of Erysipelas.*

It has been stated, on the authority of an American physician, that, in cases of erysipelas, particularly of that of the face, the application of mercurial ointment to the inflamed surface is speedily followed by beneficial results. Mr. Brodie, having had occasion to put this practice to the test of experience, found that, although its utility seemed unquestionable, a very serious inconvenience attended it,—namely, salivation. Being struck, at the same time, with the idea, that the benefit, obtained in such cases, was due, more, to the adipose substance employed than to the metallic oxide combined with it, he imagined that the external application of a simple ointment might, probably, answer the same purpose. We have his authority to state, that this idea has proved correct in several instances of erysipelas, in which the simple ointment has been applied. The patient, after its application, has experienced an almost immediate relief; and the disease appeared to go through its various stages in a more favourable manner than under ordinary circumstances.

16. *Spermatic Animalculi.*

The rage for microscopy seems again to be revived. We are by no means averse to it; for we think that every channel of inquiry, however extravagant the results, may convey some useful information to the public. The existence of animalculi in the semen of divers animals, has been asserted by some, and denied by others. Hamme and Leewenhoek, Hatzœchen and Needam, have each, in their turn, called the attention of the public to this subject. The observations of Spallanzani, by far the most accurate and philosophical, are too well known to need being repeated. Dr. I. L. Prevost, of Geneva, and Mr. Dumas, a pharmaceutical pupil, have, again, taken up this question; and have lately published a dissertation upon it, containing many curious experiments, which go to prove, in the opinion of the authors, that—1st, the existence of spermatic animalculi is unquestionable; 2d, that they are distinct, in their characters, from the *vermes infusorii*, with which they have been confounded; 3d, that they are the produce of the testicles only, and are to be found in that organ when the animal has reached the age of puberty; and, lastly, that they appear to constitute the active principle of semen, in the same manner as the globules of the blood are the indispensable elements of that fluid.—*Memoires de la Societé de Physique et d'Histoire Naturelle de Geneve,* vol. i.

17. *Tribute of Gratitude and Esteem to* Sir Gilbert Blane, Bart.

It is pleasing to have to record an act of justice to an old and highly-meritorious servant of the public,—to one who, to the arduous practice of his profession during a long series of years, has united the pursuits of science and literature,—to a gentleman who, when at the head of a public Board for the medical department of that service to which England owes its first triumphs and its first glory, in the late eventful war, exhibited that knowledge, zeal, and assiduity, which have never been disputed in the chief medical officer of the naval service of this country. In the latter capacity, Sir Gilbert Blane has ever promoted and maintained the prosperity of the medical officers under his command. As a literary man, his merits have been long known and acknowledged; while, of his qualifications, as a private member of that profession on which he confers lustre, the favourable opinion of his brethren has ever been unanimous. We feel confident, therefore, that the following correspondence will be read with satisfaction; and that our readers at large will agree in the sentiments expressed by the medical officers of the navy.

"At a meeting of a few of the senior medical officers of His Majesty's Navy, held in London, June 1820, for the purpose of taking into consideration the propriety of paying some gratifying mark of respect to Sir Gilbert Blane, Bart. for the distinguished manner in which he has employed his great talents in promoting the general interests of the medical department of the navy; and to show their sense of the successful zeal and perseverance evinced by him, while Physician to the Fleet, and Commissioner of Sick and Wounded Seamen. It was resolved, that a piece of plate, valued ONE HUNDRED GUINEAS, should be presented to him; and that A. C. HUTCHISON, Esq. late Surgeon to the Royal Naval Hospital at Deal, be authorized to act as treasurer on the occasion, and receive all subscriptions. The thanks of the meeting were given to Dr. GILLESPIE, for his conduct in the chair."

The above resolution was carried into effect in December, 1821; as will be seen from the annexed correspondence.

"*London; December 25th*, 1821.

"Sir,—A great proportion of the senior physicians and surgeons of the Royal Navy, long deeply impressed with a just sense of the extensive benefits which they, and the public service, have derived from your professional writings and eminently-distinguished acquirements, as well as from the zeal you have ever evinced in promoting the interests of the medical officers of the Royal Navy, have deputed us to present to you this piece of plate, as a small memorial of their high esteem and approbation.

"They are, at the same time, perfectly sensible, that, whatever might be the value of this mark of their esteem, it must necessarily fail to be commensurate to the merits of the acceptor, and would, at best, but feebly express their regard.

"With the highest sentiments of veneration and respect, we have the honour to be, Sir, your very grateful and humble servants,

"A. COPLAND HUTCHINSON, *Treasurer*,
"R. W. BAMPFIELD, *Secretary*."

"*To Sir Gilbert Blane, Baronet*, M.D. F.R.S.
*First Physician to the King; formerly Commissioner for Sick and Wounded Seamen, &c.*

"*London; 17th December*, 1821.

"Gentlemen,—I beg you to return my very cordial and sincere thanks to the senior physicians and surgeons of the Royal Navy, for their splendid gift, and the address which accompanies it. I should be unworthy of such a flattering distinction, were I not to feel it as one of the highest and most gratifying that could be conferred on me; being the spontaneous and liberal expression of the good opinion and good will of a class of gentlemen, whose zeal and talents as my fellow-labourers in the sacred cause of humanity, and whose devotion to the service of our King and country, it has been my lot to witness for a period of more than forty years. I only wish I could persuade myself that personal partiality may not have influenced them to over-rate my claim to so honourable a token of their approbation of my public and professional conduct. I, nevertheless, trust that I may cherish this incident, as a genuine source of delightful recollection, to the latest hour of my life: and, when that hour arrives, that I may fairly glory in this testimonial, as a sweet assurance that I have not altogether missed what has been the great end and aim of all my labours,—that of being able to reflect, in my last moments, that I have not lived in vain.

"I have farther to beg you, Gentlemen, to accept the like tribute of gratitude and esteem as I have endeavoured, so inadequately, to express towards those by whom you have been delegated, along with my best thanks for the polite manner in which you have fulfilled their kind intentions.

"Believe me, with sentiments of unfeigned regard, Gentlemen, your most faithful and most obedient servant,

"GILBERT BLANE."

"*To Alexander Copland Hutchinson, Esq. late Surgeon of the Royal Naval Hospital at Deal; and R. W. Bampfield, Esq. Surgeon, R.N.*"

**The plate bears the following inscription:**

VIRO CLARISSIMO GILBERTO BLANE, BARONETTO, S.R.S.

REGIS GEORGII IV. ARCHIATRO.

BRITTANIARUM CLASSIS OLIM MEDICO

REIQUE MEDICÆ NAVALIS

CURATORI.

SUMMÆ OBSERVANTIÆ ET REVERENTIÆ

ERGO

MEDICI ET CHIRURGI NAVALES SENIORES

D. D.

ANNO M.DCCCXXI.

[*The names of the Subscribers will be inserted in the next Number.*]

---

### BILLS OF MORTALITY,

*From December* 12, 1820, *to December* 11, 1821.

| DISEASES. | |
|---|---|
| Abscess | 88 |
| Apoplexy | 251 |
| Asthma | 694 |
| Cancer | 79 |
| Childbed | 202 |
| Consumption | 3639 |
| Convulsions | 2921 |
| Cow-pox | 1 |
| Croup | 101 |
| Diarrhœa | 5 |
| Dropsy | 769 |
| Dropsy in the Brain | 290 |
| Dropsy in the Chest | 75 |
| Epilepsy | 2 |
| Eruptive Diseases | 17 |
| Erysipelas, or St. Anthony's Fire | 23 |
| Fever | 1101 |
| Fever (Typhous) | 48 |
| Fistula | 1 |
| Flux | 5 |
| Gout | 24 |
| Hæmorrhage | 36 |
| Whooping Cough | 614 |
| Inflammation | 1309 |
| Inflammation of the Liver | 57 |
| Insanity | 222 |
| Jaundice | 100 |
| Jaw Locked | 1 |
| Measles | 547 |
| Miscarriage | 6 |
| Mortification | 145 |
| Old Age and Debility | 2535 |
| Palsy and Pleurisy | 184 |
| Rheumatism | 18 |
| Rupture | 36 |
| Scrofula | 6 |
| Small-pox | 508 |
| Sore Throat or Quinsey | 7 |
| Spasm | 42 |
| Still-born | 688 |
| Stone | 15 |
| Stoppage in the Stomach | 12 |
| Suddenly | 222 |
| Teething | 428 |
| Thrush | 78 |
| Venereal | 6 |
| Worms | 1 |
| Total of Diseases, | 18,161 |

| CASUALTIES. | |
|---|---|
| Bruised | 1 |
| Burnt | 38 |
| Drowned | 83 |
| Excessive Drinking | 1 |
| Executed* | 18 |
| Found Dead | 5 |
| Frightened | 1 |
| Killed by Falls and several other Accidents | 92 |
| Murdered | 7 |
| Poisoned | 3 |
| Scalded | 3 |
| Suffocated | 6 |
| Suicides | 32 |
| Total of Casualties, | 290 |

| | | | |
|---|---|---|---|
| Christened | Males | 13,072 | In all, 25,232 |
| | Females | 19,160 | |
| Buried | Males | 9,379 | In all, 18,451 |
| | Females | 9,072 | |

Whereof have died:

| | | | |
|---|---|---|---|
| Under Two Years of Age | 4276 | Sixty and Seventy | 1612 |
| Between Two and Five | 1793 | Seventy and Eighty | 1312 |
| Five and Ten | 904 | Eighty and Ninety | 771 |
| Ten and Twenty | 628 | Ninety and a Hundred | 150 |
| Twenty and Thirty | 1358 | A Hundred | |
| Thirty and Forty | 1817 | A Hundred and One | |
| Forty and Fifty | 1957 | A Hundred and Two | |
| Fifty and Sixty | 1872 | A Hundred and Eight | 1 |

Decreased in the Burials this year, 897.

---

* There have been executed in London and the county of Surrey, 34; of which number, 18 only have been reported to be buried within the Bills of Mortality.

### METEOROLOGICAL JOURNAL.

**By Messrs. William Harris and Co. 50, Holborn, London.**

*From December* 20, 1821, *to January* 19, 1822.

| | MOON. | Rain gauge | THERM. | | | BAROM. | | DE LUC'S HYG. | | WIND. | | ATMOSPHERIC VARIATION. | | |
|---|---|---|---|---|---|---|---|---|---|---|---|---|---|---|
| | | | 9 A.M. | MAX. | MIN. | 9 A.M. | 10 P.M. | 9 A.M. | 10 P.M. | 9 A.M. | 10 P.M. | 9 A.M. | 2 P.M. | 10 P.M. |
| Dec | | | | | | | | | | | | | | |
| 20 | | .35 | 43 | 48 | 43 | 29.27 | 29.23 | 63 | 61 | SW | SSW | Ra.&Fog | Cloud. | Rain |
| 21 | | | 45 | 49 | 42 | 29.20 | 29.35 | 58 | 60 | SW va. | SW va. | Cloudy | | |
| 22 | | | 45 | 48 | 41 | 29.49 | 29.30 | 56 | 58 | W var. | W var. | Cloudy | Fine | Cloud. |
| 23 | | .11 | 42 | 49 | 43 | 29.41 | 29.28 | 62 | 60 | WNW | SW | Fine | | Sho'ry |
| 24 | ● | .20 | 44 | 49 | 40 | 28.55 | 28.31 | 60 | 61 | SSE | ESE | Fine | Rain | |
| 25 | | | 41 | 48 | 36 | 28.53 | 28.68 | 57 | 53 | W var. | WSW | Fine | | |
| 26 | | .47 | 38 | 44 | 36 | 28.87 | 28.85 | 55 | 56 | W | WNW | Rain | | |
| 27 | | .39 | 41 | 43 | 39 | 29.01 | 28.68 | 58 | 58 | SW | S var. | Showery | Rain | |
| 28 | | .70 | 40 | 45 | 40 | 28.50 | 28.41 | 60 | 61 | SSE | S var. | Cloudy | Storm. | Fine |
| 29 | | .25 | 41 | 44 | 39 | 28.49 | 28.86 | 60 | 59 | NW | NW | Rain | Cloud. | Rain |
| 30 | | .19 | 40 | 45 | 37 | 29.25 | 29.60 | 57 | 56 | NNW | N | Rain | | Fine |
| 31 | ☾ | | 58 | 44 | 38 | 29.91 | 30.05 | 57 | 57 | NNW | NW | Fine | | Cloud. |
| Jan. | | | | | | | | | | | | | | |
| 1 | | | 39 | 44 | 34 | 29.81 | 29.67 | 58 | 57 | SSW | WSW | Cloudy | | Fine |
| 2 | | | 35 | 40 | 34 | 29.65 | 29.85 | 57 | 57 | WNW | W | Fine | | |
| 3 | | | 35 | 41 | 35 | 29.70 | 29.55 | 58 | 57 | W | SSE | Fine | Cloud. | Rain |
| 4* | | .20 | 36 | 37 | 34 | 29.18 | 29.50 | 58 | 62 | ENE | NE va. | Rain | Sho'ry | Fine |
| 5 | | | 35 | 38 | 34 | 29.86 | 30.01 | 60 | 63 | NE | NNE | Fine | | Cloud. |
| 6 | | | 35 | 37 | 32 | 30.02 | 30.09 | 64 | 65 | NE | NE | Fine | | |
| 7 | ○ | | 34 | 37 | 34 | 29.96 | 30.01 | 65 | 63 | ENE | NNE | Cloudy | | |
| 8 | | | 35 | 39 | 39 | 30.10 | 30.11 | 63 | 65 | N | NW | Fine | Cloud. | |
| 9 | | | 41 | 48 | 35 | 30.17 | 30.21 | 55 | 65 | NE | N | Cloudy | | Fine |
| 10 | | | 37 | 42 | 40 | 30.13 | 30.15 | 66 | 65 | W | W | Cloudy | | |
| 11 | | | 42 | 47 | 42 | 30.23 | 30.29 | 67 | 67 | WNW | WNW | Cloudy | | |
| 12 | | | 44 | 48 | 43 | 30.32 | 30.34 | 66 | 67 | WNW | W | Foggy | | Cloud. |
| 13 | | | 44 | 49 | 42 | 30.35 | 30.28 | 66 | 65 | W var. | WNW | Fine | Cloud. | Fine |
| 14 | | .06 | 43 | 49 | 40 | 30.26 | 30.20 | 62 | 58 | WNW | NW | Fine | | |
| 15 | ☽ | | 40 | 39 | 32 | 30.17 | 30.26 | 57 | 50 | N | N | Fine | | |
| 16 | | | 33 | 36 | 30 | 30.19 | 30.12 | 53 | 58 | NW | N | Fine | | |
| 17 | | .17 | 34 | 36 | 34 | 30.16 | 30.27 | 65 | 63 | NW | NW | Snow | Sho'ry | Fine |
| 18 | | | 36 | 46 | 35 | 30.33 | 30.38 | 67 | 63 | W | W | Foggy | | Cloud. |
| 19 | | | 38 | 46 | 42 | 30.39 | 30.34 | 66 | 65 | W | W | Cloudy | | |

The quantity of rain during the month of December, was 4.51 inches.

The late depression of the Barometer having excited the attention of many, the following results, during December, may not, perhaps, be here misplaced:

Mean height of the Barometer .................... 29.55°
Greatest elevation, occurred on the 11th, at 10 P.M. 30.35
Greatest depression, occurred on the 24th, at 10 P.M. 28.31

A Correspondent, at Hamburgh, has transmitted to us the following observations taken in that city:—Mean temperature of December, (Fahr.) 44.°1; Maximum, occurred on the 20th, 49°; Minimum, occurred on the 8th, 25°.—Also, mean height of the Barometer, 29.60°; greatest elevation, occurred on the 12th, 30.45; greatest depression, occurred on the 25th, 28.22°.

* Having (this night) changed the situation of our De Luc's Hygrometer, to where it will be subjected to more direct and undisturbed influences of the atmosphere than hitherto the indications, it is hoped, will prove additionally satisfactory.

